# Study on the equity of medical services utilization for elderly enrolled in different basic social medical insurance systems in an underdeveloped city of Southwest China

**DOI:** 10.1186/s12939-018-0765-5

**Published:** 2018-05-02

**Authors:** Rao Chen, Ning-xiu Li, Xiang Liu

**Affiliations:** West China School of Public Health, Sichuan University, No. 17 Section 3, Renmin South Road, Chengdu, 610041 Sichuan China

**Keywords:** Utilization of medical services, Basic social medical insurance, Health equity, Elderly

## Abstract

**Background:**

The equity of medical services utilization for elderly individuals enrolled in different basic social medical insurance systems holds significant meaning for social harmony against a background of demographic aging and a growing wealth gap in China. This study is to explore the equity of the three medical insurance systems in southwest China with the aim of providing recommendations for relevant policy.

**Methods:**

A total of 9600 elderly people insured through basic social medical insurance were selected and interviewed with a questionnaire. This study used a binary logistic regression model to investigate the effect of household income for medical services utilization and adopted a concentration index to measure the inequity of medical services utilization among elderly participants enrolled in different medical insurance categories.

**Results:**

Outpatient services utilization was almost identical in the different insurance systems (78.5%, 77.7% and 78.6%). There were no statistically significant differences according to income level in the Urban Employee Basic Medical Insurance (UEBMI) and Urban Resident Basic Medical Insurance (URBMI) programs, but in the New Cooperative Medical Scheme (NCMS), higher-income groups tended to utilize more services. The corresponding concentration index (CI) values were 0.0162, 0.0173 and 0.0179 respectively. The NCMS showed a lower hospitalization rate than UEBMI and URBMI (17.7% vs 24.2% and 24.9%). The higher income group utilized hospitalization more, regardless of the insurance system. The corresponding CI values were 0.0817, 0.0605 and 0.0319 respectively.

**Conclusion:**

The equity of medical services utilization for elderly exist in all three health insurance systems, in particular, the inequities in utilization of hospitalization were more severe than outpatient services. UEBMI and URBMI were better than NCMS in the equity of outpatient services. Although NCMS was more equitable than URBMI and UEBMI in terms of hospitalization, this was based on “overall low utilization of hospitalization regardless of income levels” in NCMS compared with URBMI and UEBMI. The disparities of the three basic social medical insurance systems should be eliminated. For low-income residents, specific insurance policies including reducing deductible, covering more medical service and increasing reimbursement ratio could be considered.

## Background

Over the last few decades, as China’s economic reform has sparked unprecedented economic growth, social resources have grown as well [1]. At the same time, concern for the equitable distribution of social benefits has also grown. As one of the most important social benefits, health resources have attracted considerable public attention. However, in recent years, as China’s health inequities have been increasing, public dissatisfaction has been growing [2, 3]. Health equity is the absence of systematic disparities in health between groups with different levels of underlying social advantage/disadvantage [4]. While health equity includes several important elements [5], medical services utilization, as one of these elements, was the focus of our study. The equity of medical services utilization does not mean that people with different incomes have the same rate of utilization. Instead, real equity means that utilization is not influenced by circumstances such as the ability to pay, but that people who need the services can utilize the services without financial burden [6].

Empirical evidence indicates that there is a strong correlation between income and the utilization of medical services [7, 8]. Furthermore, there is a growing income gap between rich and poor in China [9]. As a result, economic inequity is bound to cause inequity in medical services utilization [8]. To reduce inequity, the Chinese government has established a graded social medical insurance system to serves as the major source of healthcare financing and payment, which consists of three types of medical insurance. The major distinguishing features of these three schemes are that the availability of a specific medical insurance schemes depends on one’s employment status and/or residency status (urban vs. rural) [10]:Urban Employee Basic Medical Insurance (UEBMI) for the urban employed, Urban Resident Basic Medical Insurance (URBMI) for the urban unemployed resident, and the New Cooperative Medical Scheme (NCMS) for the rural population [11]. These three medical insurance schemes covered approximately 1.2 billion people (90% of the Chinese population), but each scheme operates independently, and contributions come from a variety of sources, including the central government, local governments, employers, employees, and residents [12]. For UEBMI, both employers and employees are required to contribute approximately 6 and 2%, respectively, of employees’ annual wages to the scheme. The source of the other two schemes are from individual premium contributions and subsides from central and local government. The government subsides in 2011 were 200 Yuan, individual premium contributions were varied by locations. So, the per-capita fund and annual maximum reimbursement cap were different in different schemes. In regard to the service package, compared with the comprehensive coverage of UEBMI, the other two schemes’ coverage focused on inpatient and catastrophic illness for outpatient services, while denying coverage for some basic outpatient services [10]. Table 1 contains basic information about the three social health insurance schemes in China [10, 13].

**Table 1:** Basic information about the three medical insurance schemes in China

	UEBMI	URBMI	NCMS
Inception year	1998	2007	2003
Eligible population	Urban, employed	Urban, non-employed	All rural population
Number of people insured (2013)	265 million	271 million	805 million
Source of founding (2011)	Contributory (8% of annual wage, 6% from employers, and 2% from employees)	Government subsidy (200 Yuan) and individual premium (varied by locations)	Government subsidy (200 Yuan) and individual premium (varied by locations)
Per-capita fund (2013)	2688.9 Yuan	419.1 Yuan	387.5 Yuan
Annual maximum reimbursement cap(2011)	6 times of disposable personal income (at least 50,000 Yuan)	6 times of disposable personal income (at least 50,000 Yuan)	at least 50,000 Yuan
Inpatient and outpatient services for catastrophic illness	Yes	Yes	Yes
General outpatient services	Comprehensive	Limited and varied by locations	Limited and varied by locations

Many studies have concluded that the medical insurance system plays a vital role in the equity of medical services utilization [13–16]. Additionally, they have shown that great disparities exist between people insured by different medical insurance schemes with regard to access to medical services. These disparities are part of the nature of the three systems in China, which base the level and type of insurance on individuals’ social attributes and function independently of each other, differing, for example, in aspects related to financing, reimbursement, and expansion as above [11]. Previous studies have attached great importance to comparing the three systems. For example, Meng et al. [13] and Lin et al. [17] noted that rural populations have more restricted access to medical services and show a larger financial burden than urban cohorts, mainly due to low funds for the NCMS. Furthermore, UEBMI has more comprehensive services coverage and financial protections than URBMI and the NCMS [18].

Recent years, populations are growing older in nearly all the countries of the world especially in China [19]. Ageing process could reflect some extent of socioeconomic development, for instance the health problems of the aging of population have been a great challenge to social development [20, 21]. Many studies have indicated that the elderly as a group are the greatest adult users of the health care system due to poor health [22–24]. A number of scholars have begun to explore the health inequity among the elderly. For instance, Lu et al. [25] found that health equity was at a general level and that inequity in hospitalization was significant among the elderly in Fujian province. However, to date, there has been little research on the equity of medical services utilization among the elderly categorized according to the three categories of medical insurance in China.

This study was conducted in southwestern China, in Sichuan province, which is one of China’s relatively undeveloped provinces. The aging problem is serious in Sichuan province, where the proportion of people aged over 60 was 20.02% in 2016 [26]. With this background, we conducted this study to compare the utilization of outpatient and hospitalization services among elderly individuals enrolled in the three medical insurance schemes i.e. UEBMI, URBMI and NCMS, and to measure the inequity of the utilization due to household income in the different medical insurance schemes in a city of Sichuan province in southwest China. Our study provides possible explanations for the disparity in utilization of medical services among the elderly and provides some suggestions for formulating policy that could eliminate such inequity.

## Methods

### Participants and sampling

The study used a multiple-stage cluster sampling method to randomly select the participant sample. The entire county was clustered by the government administrative geographic system (i.e., town and village). A total of 24 towns and 96 villages were randomly selected. In each community or village, about 100 households were randomly selected, resulting in 10,000 households. All family members were invited to the survey. Of the participants investigated, 9793 individuals aged 60 years and above were our study population.

### Measures

We collected data through face-to-face interviews by a three-part questionnaire. The first part covered social-demographic characteristics including gender, age, education, insurance, household income; the second part was the need for medical services including presence of physician-diagnosed chronic diseases and two-week health situation; the third part was the state of medical services utilization including physician visit within the last 2 weeks and hospitalization within the last 1 year.

The dependent variables in our study were the utilization of outpatient services in the last 2 weeks and hospitalization services within the last 1 year. The survey asked, “During the past 2 weeks, have you been sick?” Those who had illness were further asked, “Have you see a doctor for treatment?” We grouped respondents who visited the doctor as outpatient service users. The remaining individuals were classified as non-outpatient service users. Hospitalization in the last year was determined by the survey question, “Have you been hospitalized in the past year?” From this question, we categorized respondents into hospital users and nonhospital users.

### Statistical analysis

First, we used the Chi-square test to compare demographic characteristics among the elderly enrolled in different medical insurance categories. Second, binary logistic regression models were used to study the disparity in utilization of outpatient services and hospitalization services among the three cohorts; the average household income was the important independent variable we focused on.

Before the binary logistic regressions, we divided each of the categories into five levels based on the average household income (Table 2). We did not subdivide these categories according to a unified standard because there was an obvious difference between the three kinds of participants in terms of income, and the main aim of the study was to explore the internal inequities of the three categories in the insurance system.

**Table 2:** The delamination standard of income among the three categories (Yuan)

Income grade	UEBMI	URBMI	NCMS
I	≤2500	≤1200	≤350
II	2501–3520	1201–2000	351–800
III	3521–4500	2001–2800	801–1370
IV	4501–5600	2801–4000	1371–2500
V	>5600	>4000	>2500

For outpatient, the dependent variable was the utilization of medical services in the last two weeks if the person had illness, and the independent variables included gender, age, education, the status of chronic diseases and the average household income.

For hospitalization, the dependent variable was the utilization of hospitalization services, and the independent variables were the same as the outpatient services except that the two-week health situation was added.

All the variables used in these binary logistic regressions are summarized in Table 3.

**Table 3:** Summary of the variables in these binary logistic regressions

Variables	Type of variable	Measurement
Dependent variables		
utilization of outpatient services	Categorical	No = 0; Yes = 1
utilization of hospitalization services	Categorical	No = 0; Yes = 1
Independent variables		
gender	Categorical	Male = 1; Female = 2
age	Categorical	60~ 74 = 1;75~ = 2
education	Categorical	No formal education = 1; Primary school = 2; Junior high school or higher = 3
chronic diseases	Categorical	No = 0; Yes = 1
two-week prevalence^a^	Categorical	No = 0; Yes = 1
household income	Categorical	Grade I = 1; Grade II = 2; Grade III = 3; Grade IV = 4; Grade V = 5

^a^only in the binary logistic regression for hospitalization

Finally, we applied the concentration index (CI) to measure the equity in utilization of medical services among the three cohorts.

The concentration curve provides a means of assessing the degree of income-related inequity in the distribution of a health variable [27], which plots the cumulative percentage of the population, ranked by economic state (x-axis), against the cumulative percentage of the health variable (y-axis). If everybody has the same value of the health variable regardless of economic state, the concentration curve will coincide with the 45-degree line which is called the line of equity. If the concentration curve lies above (below) the line of equity, it means that poorer people have higher (lower) values of the health variable.

The CI is defined as twice the area between the concentration curve and the line of equity. The index is negative when the curve lies above the line of equity. On the other hand, the index takes a positive value when the curve lies below the line of equity. The index can be calculated by the following equation:

1 $$ \mathrm{C}=2/\upmu \mathrm{cov}\left({\mathrm{h}}_{\mathrm{i}},{\mathrm{r}}_{\mathrm{i}}\right) $$

where h_i_ is the health status of the ith individual, r_i_ is the fractional rank of the ith individual in terms of economy and μ is the mean of the health status.

The CI value ranges from − 1 to 1, and the value represents the level of the equity. A positive (negative) index suggests the health variable concentrated among the rich (poor). The result indicates greater equitability when the value approaches 0 [28].

In this study, we calculated the CI of the utilization rate to explain the underlying equity. However, the utilization rate can be influenced by various factors, so before the calculation, we standardized the rate to keep the other factors, except for the economy, at the same level to ensure that the rate was influenced by the economy only. Our specific methods are outlined below:

First, we built the equation based on the binary logistic regression models:

2 $$ \ln \left(\mathrm{P}/1\hbox{-} \mathrm{P}\right)={\upalpha}_0+\sum {\upbeta}_{\mathrm{i}}{\mathrm{x}}_{\mathrm{i}}+{\upbeta}_{\mathrm{j}}{\mathrm{x}}_{\mathrm{j}} $$

In the equation, P represents the rate; x_i_ represents the other factors influencing the rate, such as gender, age and so on; and x_j_ represents the economy. We substituted x_i_ with the mean value to minimize their effects and maximize the influence of economy.

Finally, we substituted standardized rates into this formula (1) to get the corrected CI.

All data management and statistical analyses were performed with SPSS 21.0 (descriptive analyses and binary logistic regressions) and SAS 9.4 (concentration index).

## Results

### Descriptive analysis

A total of 9793 adult participants over age 60 participated. Of these, 9600 participants (1385 in UEBMI, 687 in URBMI, and 7528 in the NCMS), or 98.03%, were insured by the basic social medical insurance. Table 4 presents the descriptive statistics of variables used in this study for those who were insured through the basic social medical insurance system. As shown in the table, there were demographic differences (gender, age, education, household income) between the participants in the three categories. Additionally, the average household monthly income was degressive in the sequence of UEBMI, URBMI, and the NCMS, and individuals who were enrolled in UEBMI demonstrated greater medical needs.

**Table 4:** Summary statistics among the three cohorts enrolled in different medical insurance systems

Variables	UEBMI (*N* = 1385)	URBMI (*N* = 687)	NCMS (*N* = 7528)	*χ*^*2*^/F	*P*
Gender				57.177	< 0.001
male	773(55.8%)	263(38.3%)	3685(49.0%)		
female	612(44.2%)	424(61.7%)	3843(51.0%)		
Age, year				11.255	0.004
60~ 74	1008(72.8%)	539(78.5%)	5761(76.5%)		
75~	377(27.2%)	148(21.5%)	1767(23.5%)		
Education				1715.927	< 0.001
no formal education	98(7.1%)	186(27.1%)	2738(36.4%)		
Primary school	478(34.5%)	344(50.1%)	3902(51.8%)		
Junior high school or higher	809(58.4%)	157(22.8%)	888(11.8%)		
Having chronic diseases	914(66.0%)	417(60.7%)	4602(61.2%)	11.980	0.003
Two-week prevalence^a^	717(51.8%)	309(45.0%)	3166(42.1%)	45.378	< 0.001
Average household income (Yuan)/month	4202.68	2927.31	1556.15	1363.467	< 0.001

^a^The two-week prevalence was the prevalence before the study within two weeks

The indicators of the utilization of medical services are presented in Table 5. A total of 78.5% of individuals said they see a doctor when they feel physically ill, and there was no significant difference among the three different insurance systems. However, it was clear that those enrolled in the NCMS had lower hospitalization rates. In regard to medical expenses, compared with UEBMI and URBMI, the expense of participants enrolled in the NCMS was lower. However, in reality, those enrolled in the NCMS generally incurred more cost than their UEBMI counterparts when they utilized the hospitalization medical services, due to their lower rate of reimbursement.

**Table 5:** The indicators of the utilization of medical services among the three categories

Variables	UEBMI	URBMI	NCMS	Average	*χ*^*2*^/F	*P*
Two-week visiting rate among ill people (%)	78.5	77.7	78.6	78.5	0.160	0.923
Per outpatient expense (Yuan)	469.71	328.09	290.01	323.90	5.985	0.003
Hospitalization rate (%)	24.2	24.9	17.7	19.2	46.817	< 0.001
Per inpatient expense (Yuan)	9486.19	9220.78	5958.10	6902.62	18.047	< 0.001
Inpatient expenses by self (Yuan)	2995.28	3825.72	3464.61	3412.10	0.823	0.439
Rate of reimbursement (%)	68.42	58.51	41.85	49.43	14.174	0.001

### Binary logistic regressions analysis

We performed binary logistic regressions for the utilization of outpatient and hospitalization services to investigate the influence of income on utilization. Table 6 shows the results of income in outpatient services. In regards to utilization of outpatient services, as the table shows, there was no statistical disparity among the different income levels in UEBMI and URBMI. However, in the NCMS, there was a significant indication that those with higher incomes tended toward higher utilization.

**Table 6:** Binary logistic regression analyses for utilization of outpatient services

System	Income grade	*B*	*P*	OR^a^(95%CI)
UEBMI	I			1.000
	II vs I	−0.018	0.950	0.982(0.559~ 1.725)
	III vs I	0.423	0.159	1.526(0.847~ 2.748)
	IV vs I	0.283	0.349	1.327(0.735~ 2.395)
	V vs I	0.289	0.340	1.336(0.738~ 2.418)
URBMI	I			1.000
	II vs I	0.155	0.712	1.167(0.514~ 2.653)
	III vs I	0.024	0.956	1.024(0.433~ 2.421)
	IV vs I	0.229	0.577	1.257(0.562~ 2.813)
	V vs I	0.377	0.449	1.458(0.549~ 3.874)
NCMS	I			1.000
	II vs I	0.080	0.536	1.083(0.842~ 1.393)
	III vs I	0.145	0.297	1.156(0.881~ 1.517)
	IV vs I	0.287	0.035	1.332(1.020~ 1.741)
	V vs I	0.352	0.018	1.422(1.063~ 1.902)

^a^OR: Odds Ratio

From Table 7, we can see that people with higher incomes showed a greater likelihood of utilizing hospitalization services than poorer people, regardless of the insurance system. In addition, we found that the odds ratio of UEBMI tended to be larger as the income increased, while this value was relatively stable in the NCMS.

**Table 7:** Binary logistic regression analyses for utilization of hospitalization services

System	Income grade	*B*	*P*	OR^a^(95%CI)
UEBMI	I			1.000
	II vs I	0.443	0.011	1.557(1.105~ 2.195)
	III vs I	0.424	0.018	1.529(1.075~ 2.174)
	IV vs I	0.586	0.001	1.796(1.283~ 2.514)
	V vs I	0.613	0.001	1.846(1.292~ 2.636)
URBMI	I			1.000
	II vs I	0.634	0.043	1.885(1.021~ 3.480)
	III vs I	0.646	0.050	1.907(1.001~ 3.635)
	IV vs I	0.796	0.008	2.216(1.233~ 3.981)
	V vs I	0.468	0.179	1.597(0.807~ 3.163)
NCMS	I			1.000
	II vs I	0.194	0.046	1.215(1.003~ 1.470)
	III vs I	0.235	0.022	1.265(1.035~ 1.547)
	IV vs I	0.194	0.048	1.215(1.002~ 1.473)
	V vs I	0.245	0.018	1.278(1.042~ 1.566)

^a^OR: Odds Ratio

### Equity analysis

Table 8 provides the results of using CI to measure the inequity of medical services utilization among the different kinds of medical insurance. First, the results indicated that all the CI values were positive and that the CI for hospitalization was larger than that for outpatient services. Moreover, the CI of the NCMS was larger than those of the two other medical insurance systems in terms of outpatient services, while the CI of hospitalization yielded precisely the opposite result.

**Table 8:** The concentration index results among the three cohorts

Income grade	Treatment standardized rate (%)			Hospitalization standardized rate (%)		
	UEBMI	URBMI	NCMS	UEBMI	URBMI	NCMS
I	74.04	70.19	71.91	14.72	13.52	13.30
II	73.69	73.33	73.50	21.19	22.76	15.70
III	81.32	70.69	74.75	20.87	22.97	16.25
IV	79.10	74.75	77.33	23.67	25.73	15.70
V	79.20	77.44	78.45	24.16	19.98	16.38
CI	0.0162	0.0173	0.0179	0.0817	0.0605	0.0319

## Discussion

The present study showed that the coverage of medical insurance was 98.0%, which indicates that China has achieved much in the establishment of basic social medical insurance. However, there are still some people who remain uninsured among a large population base, which indicates a worthy goal to strive for.

In our investigation, there was no fundamental difference among the elderly under the three kinds of medical insurance in the utilization of outpatient services if they were ill. The treatment rates were approximately 78%, it could be because the expense was affordable for the elderly. Additionally, we found that members of the NCMS had lower outpatient costs than members of the two other systems. The reason may be that the members of NCMS prefer to seek medical services in primary health care institutions where the expense is lower [10, 29].When comparing the equity of the three medical insurances, we found that there was no significant difference in the utilization of outpatient service between different income levels in UEBMI and URBMI. However, in the NCMS, there was a tendency for the higher income group to utilize more services. This indicates that the equity of UEBMI and URBMI is better than NCMS. As we know, the NCMS are mainly targeted at hospitalization and the critical diseases of outpatient, and the benefit packages for medical services are not as munificent as those of UEBMI, and the enrolled have to pay for most outpatient fees personally [11, 30]. Although the expense of outpatient service was relative lower, but there were still some low-income population in NCMS who couldn’t easily afford it [31].

As far as hospitalization is concerned, the results indicated that the higher income group utilized hospitalization more, regardless of the insurance system. Thus, it can be speculated that inequity is widespread in the utilization of hospitalization. Additionally, we found that the range of hospitalization standardized rate among different income levels in the NCMS was between 13.30% and 16.38%, and the disparity was about 3%, which was significantly smaller than URBMI and UEBM (about 10%). Interestingly, this indicated that the NCMS was more equitable than URBMI and UEBMI in terms of hospitalization. This was verified by comparing the value of CI. And the CI of NCMS was smallest among the three systems. However it should be noted that the equity in NCMS was based on the overall low utilization of hospitalization regardless of income levels. This also suggested that the economic burden of hospitalization was considerable for NCMS cohort [31]. As mentioned in Table 5, the reimbursement ratio of the NCMS was lower than for the other two systems. Furthermore, we found that the hospitalization expenses of individuals in the NCMS were higher than those of participants with UEBMI, although the actual cost of the NCMS was lower.

The CI results clarify this inequity further. All the CI values we calculated were positive, which means that the richer participants had more utilization than poorer participants, whatever the medical system and the medical services. In addition, the CIs of hospitalization were larger than those of outpatient services. This result indicates that the inequity in hospitalization is more serious than for outpatient services, and the main reason is that the expenses of hospitalization are higher and thus income is crucial in deciding utilization.

## Conclusion

We found that inequities still exist in all three health insurance systems, in particular, the inequities in utilization of hospitalization were more severe than outpatient services. When comparing the equity of the three medical insurances, UEBMI and URBMI were better than NCMS in the equity of outpatient services. NCMS was more equitable than URBMI and UEBMI in terms of hospitalization. However, the equity of hospitalization in NCMS was based on the “overall low utilization of hospitalization regardless of income levels”. Thus, basic social medical insurance can be further improved to make sure vulnerable groups utilize the medical services better. A comprehensive approach to reduce the inequity of the three basic social medical insurances should focus on the following factors: 1) Covering more basic outpatient services in the medical insurances, especially for NCMS; 2) raising the reimbursement rate of hospitalization for the three basic social medical insurance systems, especially for NCMS; 3) Eliminate the disparities of the three basic social medical insurance systems under the different health insurance plans. 4) For low-income residents, specific insurance policies including reducing deductible, covering more medical service and increasing reimbursement ratio could be considered.

## Abbreviations

CI: concentration index
NCMS: New Cooperative Medical Scheme
UEBMI: Urban Employee Basic Medical Insurance
URBMI: Urban Resident Basic Medical Insurance
